# LncRNA NALT interaction with NOTCH1 promoted cell proliferation in pediatric T cell acute lymphoblastic leukemia

**DOI:** 10.1038/srep13749

**Published:** 2015-09-02

**Authors:** Yaping Wang, Peng Wu, Rufeng Lin, Liucheng Rong, Yao Xue, Yongjun Fang

**Affiliations:** 1 Department of Hematology and Oncology, Nanjing Children’s Hospital Affiliated with Nanjing Medical University, Nanjing 210008, China

## Abstract

Long non-coding RNA (lncRNA) was referred to be participating in various malignant tumors. Location based analysis of the mechanism in lncRNA and genes have been highly focused. In this study, we reported that lncRNA named NALT which was located near NOTCH1 within 100 bp away. We confirmed that up-regulation of NALT associating with NOTCH1 in human samples. Increased expression of NALT dramatically promoted cell proliferation in cell lines via CCK8 assay and EDU stain. Further xenograft tumor also indicated the growth inducing affection of NALT while could be partial reversed by GSI. Besides, through sorting the side-population cells in T ALL cells treated with NALT shRNA could decrease percentage of SP cell which companied by the down-regulation of NOTCH1. Gal4-λN/BoxB reporter system revealed that the nuclear located NALT could function as a transcription activator which caused an activation of NOTCH signal pathway as confirmed by western blot. Taken together, we found a neighbor of NOTCH1, Lnc-RP11-611D20.2 (named NALT) which could regulate the NOTCH1 signal pathway through *ci*s-regulation. This founding may trigger a comparable development of diagnosis or novel molecularly-directed therapies.

T cell acute lymphoblastic leukemia (T ALL) is a neoplastic disorder that occurs in 15% of pediatric and 25% of adult acute lymphoblastic leukemia and is generally associated with unfavorable clinical features and aggressive biologic behavior such as higher risk for primary resistant disease, centre nervous system relapse never early relapse^1^,^2^,^3^. The prognosis of T-ALL in children has improved in recent years due to the novel and heavy chemotherapy approaches^4^; thus, it remains necessary for discovery of new molecular diagnosis assay or target spot for therapy to improve its prognosis a lot.

Recently, researchers have found that NOTCH1 activity increased extremely high in most T ALL patients^5^,^6^. Activation of the NOTCH signaling pathway confers benefits to the cell a strong growth advantage, due to the fact that NOTCH1 could directly control key regulators in cell proliferation^7^. Further analysis has found that the activation of NOTCH signal pathway mainly occurred in the subsets of stem like cells^8^,^9^,^10^. The mammalian NOTCH family is comprised of four NOTCH receptors encoded by NOTCH1, 2, 3, 4 and with five ligands including Jagged-1, Jagged-2, Delta-like-1 (DLL1), Delta-like-3 (DLL3) and Delta-like-4 (DLL4)^11^,^12^. Notch signaling is initiated by receptor-ligand interaction, which leads to the activation of NOTCH intracellular cytoplasmic domain (NICD) which inducing the promotion the transcription of genes downstream in the NOTCH pathway^13^,^14^. Previous studies have successes to suppress the activation of Notch signaling in T-ALL via the administration of gamma-secretase inhibitors (GSI), which block cleavage of Notch receptors^15^,^16^.

Currently, researches are focusing on the mechanism of long non-coding RNA (lncRNA) in many malignant diseases and have discovered that various crucial lncRNAs playing important roles in the pathogenesis of solid tumors^17^; however, the function of lncRNAs implicated in ALL or other hematological system cancers remains poorly described. Exploring the underlying mechanism of lncRNAs will be critical to recognizing the contribution of them in biological processes involved in ALL. A published study presented a new mechanism of potential target gene of lncRNA based on a location distance of a certain lncRNA and the neighbor protein coding genes and was named as Flank10k^18^. The Flank10k in detailed mean that lncRNA might regulate especially a *cis*- regulatory with a neighbor gene within the distance of 10000 bp in the chromosome location^19^,^20^. This hypothesis has been confirmed by various studies. For example, Rinn JL, *et al*. revealed that HOTAIR, a 2.2 kilobase ncRNA residing in the HOXC locus, interacts with Polycomb Repressive Complex 2 (PRC2) and is required for PRC2 occupancy and histone H3 lysine-27 trimethylation of HOXD locus^21^.

Interestingly, we found that a neighbor of NOTCH1, an lncRNA entitled RP11-611D20.2 which was located specifically in the up-stream of NOTCH1 with nearly 400 bp away from NOTCH1. We supposed that this lncRNA might associate with NOTCH1 based on the Flank10k theory. After we confirmed the aberrant expression level of RP11-611D20.2 in pediatric T ALL patients, we named it as NOTCH1 associated lncRNA in T ALL (NALT) and conducted the further experiment to exploring the potential role of the NALT in the pathogenesis of T ALL.

## Results

### Increased expression level of NALT associated with NOTCH1 in pediatric T ALL

**Figure 1:**
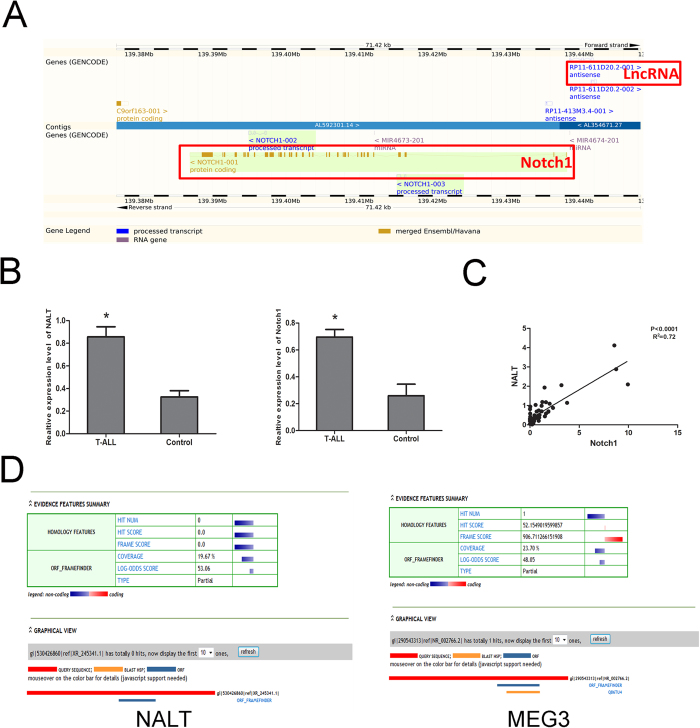
NALT was up-regulated with a high correlation with NOTCH in children T ALL. (**A**): Schematic figure presenting the location relationship of NALT and NOTCH1 in Chromosome 9. The NALT and NOTCH1 were highlighted with red box. (**B**): The up-regulation of NALT and NOTCH1 mRNA in patients bone marrow samples (n = 20) by comparing with control samples (n = 10). Data was presented with mean ± SEM. C: Pearson correlation showed a positive correlation between expression levels of NALT and NOTCH1 with a P < 0.0001, R^2^ = 0.72. D: Coding Protein Calculator (http://cpc.cbi.pku.edu.cn/) was employed to examine the protein coding ability of NALT. MEG3 was regarded as a positive control (**indicated p < 0.01).

According to the bioinformatics analyses, NALT was located in the upstream of NOTCH1, within a spacing of 10 kb (Fig. 1A). We first detected the expression level of NALT and NOTCH1 in the bone marrow of 20 T ALL children and 10 age matched volunteers as control. The up-regulation of both NALT and NOTCH1 was obtained in patients (Fig. 1B). In addition, we analyzed the correlation of NALT and NOTCH1 in patients. The Pearson correlation analysis indicated that NALT was correlated with the increased level of NOTCH1 (Fig. 1C).

Since NALT has not been reported in human disease, we conducted the experiment to confirm non protein-coding feature through bioinformatics software. The Coding Protein Calculator (http://cpc.cbi.pku.edu.cn/) was used to examine the protein coding ability of NALT. As compared with MEG3, a well-known non-coding RNA, NALT was more likely to be the non-coding RNA (Fig. 1D).

### NALT promoted cell proliferation *in vitro* and *in vivo*

**Figure 2:**
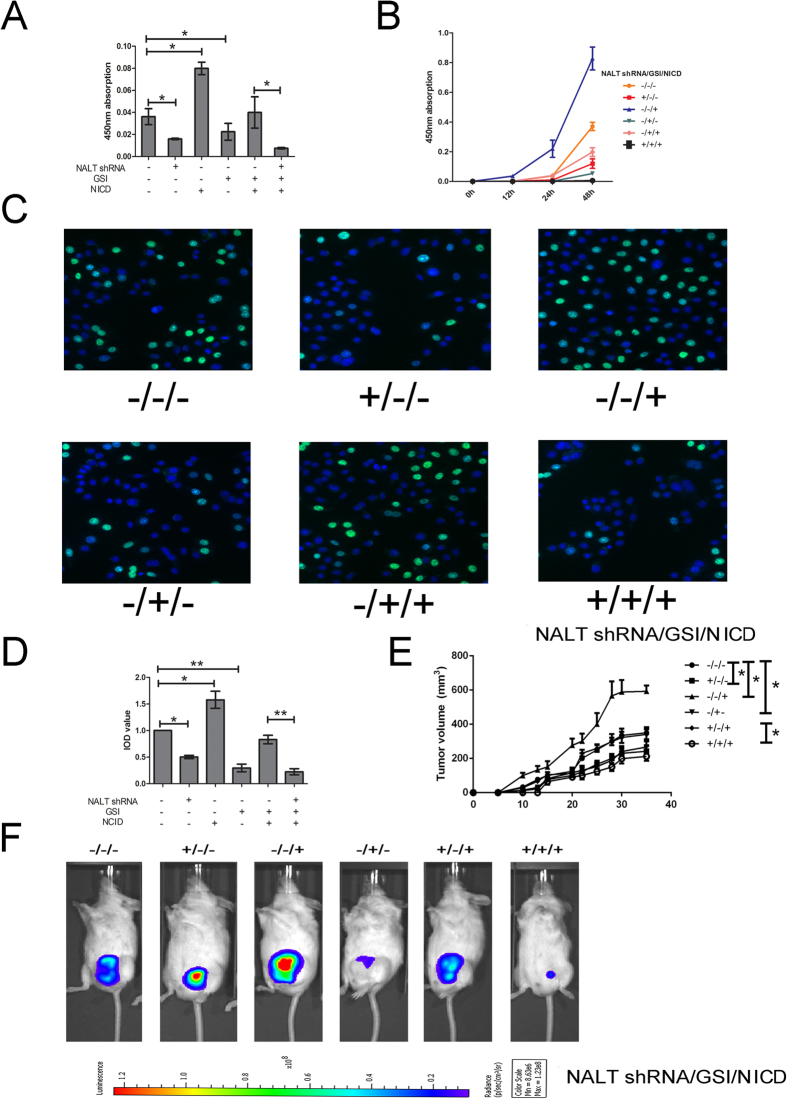
NALT promoted cell proliferation *in vitro* and *in vivo*. (**A**): The CCK8 detection in cells treated with NALT shRNA, GSI, NICD and the corresponding control after cells treated for 24h. Absorbance at 450 nm was presented as the mean ± SEM. (**B**): The absorbance was detected in 0h, 12h, 24h and 48h. (**C,D**): The EDU stain also performed in cells treated as described above with a magnification of 200. The integral optical density value of cells treated with control plasmids was normalized to 100%. E: subcutaneous injection in NOD/SCID mice with Jurkat stably expressed with NALT shRNA, GSI, NICD or control (n = 5). The volume of each tumor was calculated as the length × width^2^ × 0.5 nearly every 3 days. F: Mice with established tumors were imaged every 7 d in different groups by IVIS Lumina II system; shown are images taken at day 25. Data presented as the mean ± SEM (*indicated P < 0.05, **indicated p < 0.01).

To detect the potential function role of NALT on T ALL cells, we employed two T ALL cell lines to explorer the experiment. We first confirmed the RNA expression of NALT in Jurkat cells, based on this; we further applied the shRNA targeting NALT by lentivirus to knockdown the expression the NALT *in vitro* (Supplementary Figure S1A). Besides, to investigate whether NALT could interact with NOTCH signal pathway, we also applied GSI which was known as the inhibitor of the intracellular domain of Notch. The rescue group was also participated by the overexpression of the NICD. As presented in Fig. 2A,B, CCK8 proliferation assay were used to detect the proliferation of cells. We found that NALT shRNA could induce the proliferation suppression as compared with the mock group which was consisted with the affection of GSI. On the contrary, the NICD treated cells could partially reversed the affection caused by the decreased of NALT shRNA. Cells co-treated with NICD, GSI and NALT shRNA could induced a remarkable decreasing level of proliferating cells which indicated that the NALT might interact with NOTCH1 or influence the function of NICD induced cell proliferation. To validate this affection, we conducted a time gradient assay by CCK8 detection. The same results was obtained in cells incubated after 24 h while the affection was amplified detecting in 48 h. Further validation was conducted by using the antisense oligonucleotides (ASO) targeting NALT was consistence with the results above (Supplementary Figure S1D).

The EDU stain assay was also conducted to investigate the percentage of proliferating cells. The results demonstrated the same affection with CCK8 assay that NALT shRNA could suppressed the percentage of growing cells and could be remedied by the participating of NICD (Fig. 2C,D).

The *in vivo* tumor growth activity assay on T-leukemia was further evaluated in a murine xenograft model. Only the subcutaneous inoculation of Jurkat cells into NOD/SCID mice resulted in a tumor formation at the site of injection in all mice. The sizes of tumors formed in mice treated with NALT shRNA were significantly smaller than those of the control and co-treated group with NALT shRNA and NICD which was similar with cells treated with GSI. Consistent with *in vitro* study, cells harbored NICD promoted tumor growth comparing with the mock group (Fig. 2E,F and Supplementary Figure S1E).

### NALT targeting side population cells with the interaction of NOTCH1

**Figure 3:**
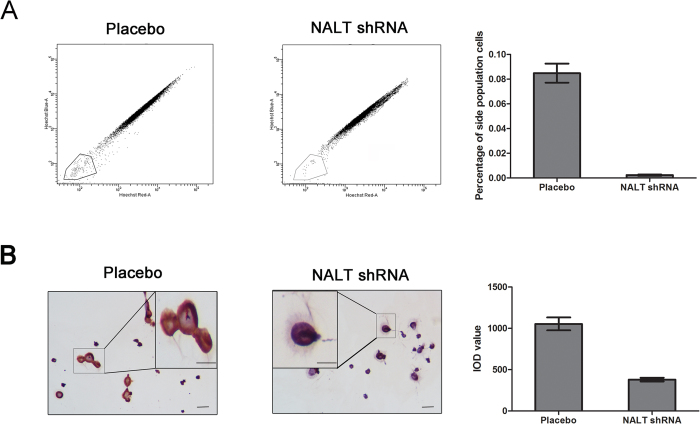
NALT targeting side population cells with the interaction of NOTCH1. (**A**): The reduction in the SP after NALT shRNA treatment detected by flow cytometry (**B**): Immunocytochemistry on cytospins of sorted cells showed that all the down-regulated NOTCH1 in SP cells treated with NALT shRNA. Scale bar: 200 mm; magnification scale bars 100 mm.

Subsequent studies have found that NOTCH signaling contributes toward the maintenance of cancer stem cells (CSCs)^22^. Side population (SP) cells are a rare subset of cells with a stem-like feature^23^. The SP phenotype has been proven to be invaluable for stem cell isolation in the absence of definitive cell surface markers^24^. Thus, to further investigate the function role induced by the aberrant expression level of NALT and to explore whether the interaction of NALT and NOTCH1 could affect the distribution of stem-like cells, we conducted the experiment to examine the proportion of SP cells in Jurkat cells in different groups. As a control, ABC transporter activity was inhibited using verapamil hydrochloride. In untreated samples, about 5% of SP cells were positive stained. After cells treated with NALT shRNA, less than about 1% of SP cells were detected, thus documenting that NALT might target the SP cell (Fig. 3A).

To further confirmed the loss of SP cells in NALT shRNA treated cells; we sorted the SP cells and conducted the ICC assay to detect the expression of NOTCH1 in SP cells. The stain presented that alone with the decreased number of SP cells, the NOTCH1 was also down-regulated in those cells (Fig. 3B) indicating that NALT targeting side population cells with the interaction of NOTCH1.

### NALT activated the transcription of NOTCH1

**Figure 4:**
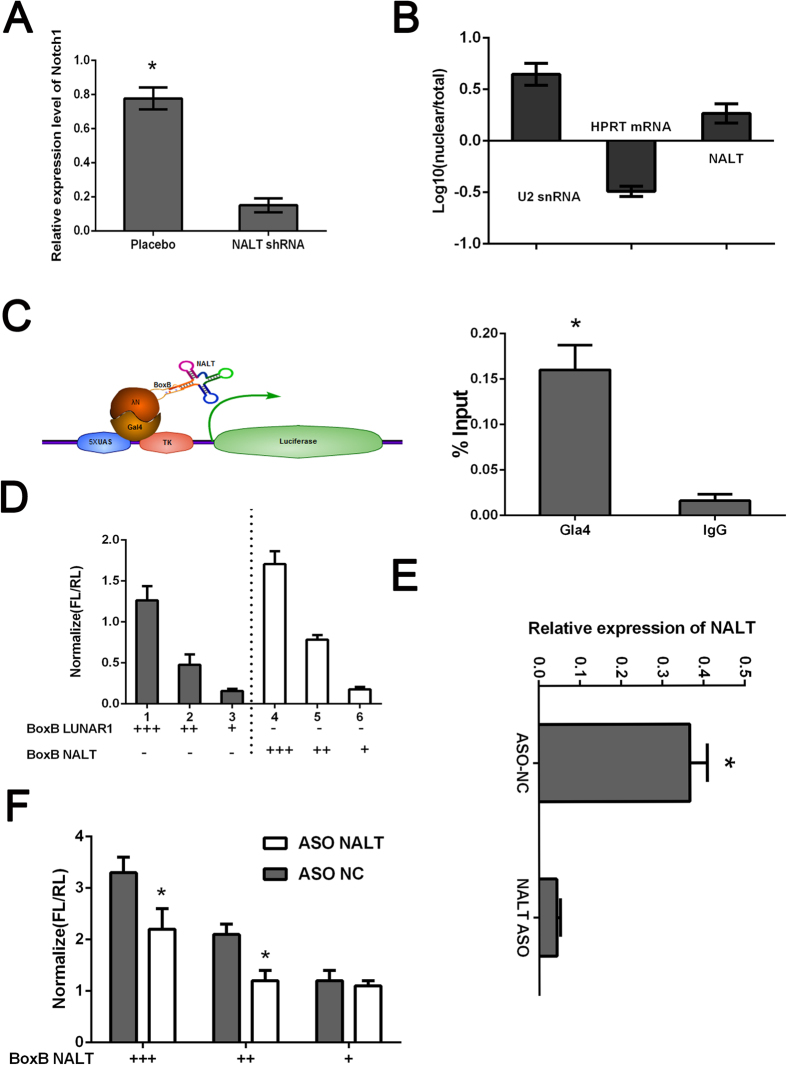
NALT promoted NOTCH1 transcription. (**A**): The mRNA expression of NOTCH1 in cells treated with NALT shRNA. (**B**): Subcellular localization of NALT in cells. HPRT was used as the control for cytoplasmic expression and U2 for cytonuclear expression. (**C**): Schematic figure for Gal4-λN/BoxB reporter system. The binding of Gal4-λN fusion was confirmed by ChIP. (**D**): Luciferase reporter activity in experiments where BoxB-tagged NALT (right) or LUNAR1 (left) were cotransfected with Gal4-lN. E: qPCR following ASO knockdown of NALT in luciferase assay. F: Reporter assay showing relative reporter gene activity when BoxB-NALT was cotransfected with either control or ASO targeting NALT. Data was normalized to Ranilla luciferase. Data presented as the mean ± SEM. *indicates p value < 0.05.

Based on the results above, we still confusing about the detailed mechanism of NALT on regulating NOTCH1. It is not clear if this effect is due to decreased NOTCH1 transcription or by post translational regulation of NOTCH1. We first applied the RT-PCR assay to detect the mRNA level of NOTCH1 in NALT depleted cells. As presented in Fig. 4A, we found that the mRNA expression of NOTCH1 was also decreased and could not rescued by over-expression of NALT indicating that NALT might regulated NOTCH1 in a *cis*-pattern (Supplementary Figure S1B,C). The further subcellular location of NALT was conducted for validation. The subcellular location of NALT was applied by using SurePrep™ Nuclear/Cytoplasmic RNA Purification Kit according to the manufacturer’s protocol. HPRT was used as the control for cytoplasmic expression and U2 for cytonuclear expression. We found that NALT was obviously existed in both cytoplasm and nucleus, and the expression of NALT in the nucleus was higher than that in the cytoplasm (Fig. 4B). Further Gal4-λN/BoxB reporter system was employed. The binding of Gal4-λN fusion was confirmed firstly (Fig. 4C). Luciferase activity after co-transfection of the system indicated that tethering NALT to this reporter gene could stimulate transcription of the reporter to a similar degree as LUNAR1 indicating that NALT could function as a transcription activator (Fig. 4D). Further ASO targeting NALT was designed (Fig. 4E), we found that the activation affection was significantly reduced comparing to control group (Fig. 4F).

**Figure 5:**
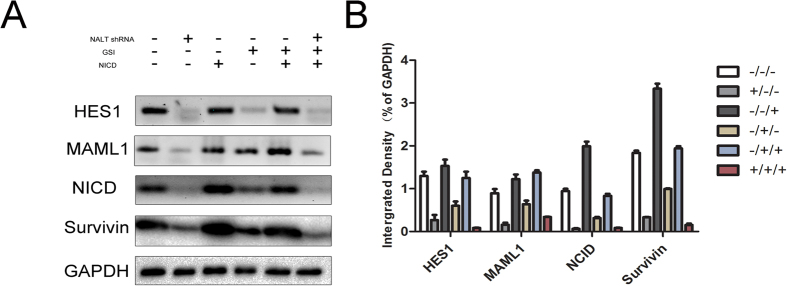
Decreased NALT suppressed the NOTCH1 signaling pathway. (**A**): Western blot was applied to confirm whether the aberrant suspension of NOTCH signal pathway was induced by the loss of NALT. HES1, MAML1 and Survivin were selected to determine the aberrant expression of Notch signal pathway. GAPDH was used as a loading control. All gels have been run under the same experimental conditions. B: Integral optical density (IOD) was calculated for each band. Data were presented as the mean ± SEM. *indicates P < 0.05.

Next, we investigated the expression levels of NOTCH pathway components in NALT knock-down cells with the participating of GSI and/or NICD. We analyzed the expression levels of HES1, MAML1, NICD and Survivin in the cells treated as described above. There was a decrease in the protein levels of the NOTCH components as a result of the knock-down of NALT. However, this down-regulated could partially be reversed by NICD and could be aggravated by GSI (Fig. 5A,B).

## Discussion

ALL has been the leading cause of cancer-related deaths in children and adolescents over four years old now. More than 10,000 new cases of childhood ALL are reported each year in China^25^,^26^. The environmental factors, such as suffering from chemical, physical, and biological agents’ exposure as well as genetic factors are considered as risk factors for ALL^27^,^28^. The discovery detailed mechanism of key factor involved in the pathogenesis of ALL provided a new approach for the development of new diagnosis method and intervening measure. For instance, the fusion gene BCR-ABL in children ALL was a millstone improvement in the diagnosis, target therapy towards the fusion genes , which is the world hottest and focal point of the tumor treatment^29^.

Recent study has been identified that lncRNA play important roles in the development in various human malignant tumor such as hepatocellular carcinoma, prostate cancer, gastric cancer and hematological system cancers^17^,^30^,^31^,^32^. They found that could regulate gene expression through modulation of chromatin remodeling, controlling of gene transcription, post-transcriptional mRNA processing, protein function or localization, and intercellular signaling. The detailed mechanism of lncRNA is still under exploiting based on the discovery that lncRNAs were involved in a network which might be modified epigenetically including methylation, ubiquitination, or miRNA-induced regulation^33^.

According to the bioinformatics analyses recently confirmed by several researchers, we conducted our exploration due to the neighborhood relation of NALT and NOTCH1, a well known oncogene in the pathogenesis of children T ALL^34^. However, single compound therapies almost invariably lead to resistance. Therefore, a deeper understanding of NOCTH signaling pathway in normal maturation and in NOTCH1 activated T-ALL could yield novel insights that could make treatment more effective.

In our study, we firstly confirmed the aberrant expression of both NALT and NOTCH1 in children suffering from T ALL. Since NOTCH signaling pathway plays multiple roles in hematopoiesis and is very essential for the establishment of definitive hematopoiesis through the generation of hematopoietic stem cells, as well as for their subsequent differentiation in an expanding number of blood cell types^35^,^36^. The more importantly, GSIs that block S3 cleavage of the receptor and subsequent release of the ICN are the subject of intensive investigation as novel drugs to combat T-ALL^37^. Our study investigated the new factor involved in the NOTCH-dependent regulation in T ALL. Through the CCK8 and EDU assay *in vitro*, we found function of the NOTCH-dependent lncRNA in cell proliferation which supporting an important role for the lncRNA in NOTCH-associated T-cell biology. In addition, the recently identified lncRNA LUNAR1 was present amongst the most robustly Notch regulated long non-coding RNAs in other sets. LUNAR1 was identified to be required for efficient T-ALL growth as a consequence of its role in enhancing IGF1R mRNA expression to sustain IGF1 signaling^38^. As a prelude to assigning functional annotation to NALT in this study, we applied the xenograft by using T ALL cell lines on NOD/SCID mice in which functions are confirmed as well as the experiment *in vitro*. To further investigate the detailed mechanism of NALT and NOTCH1, we examined the SP cells harbored the “stem like” feature. The results provided strong evidence that the NOTCH1-dependent regulation by NALT might occurred in the stem cells in the development of T ALL. Luciferase activity after co-transfection of the system indicated that tethering NALT to this reporter gene could stimulate transcription of the reporter to a similar degree as LUNAR1 indicating that NALT could function as a transcription activator leading to the activation of ICN in cytoplasm as well as the NOTCH signal based on the up-regulation of HES1, MAML1 and Survivin.

In conclusion, we found a neighbor of NOTCH1, Lnc-RP11-611D20.2 (named NOTCH1 Associated Long non-coding RNA in pediatric T ALL, NALT), a long non-coding RNA of 546nt length located in the upstream of NOTCH1 within a spacing of 10 kb. According to the Flank10k prediction, we aimed to investigate whether this lncRNA could regulate NOTCH1 by functioning as a transcription activator which caused the activation of NOTCH signal pathway. This founding may trigger a comparable development of novel molecularly-directed therapies that may improve patients’ prognosis.

## Methods

### Patient samples

Study data were obtained from 20 patients who presented between September 2013 and October 2014 at The Nanjing Children Hospital Affiliated to Nanjing Medical University (Nanjing, China). Informed consent for bone marrow analysis was obtained prior to surgery. This study was approved by the Institutional Ethics Committee of Nanjing Medical University, and it was performed in accordance with government policies and the Helsinki Declaration. Experiments were undertaken with the understanding and written consent of each subject.

### Cell lines, plasmids and reagents

T-lymphoma cell lines were available from American Type Culture Collection (Manassas, VA, USA). Cells were maintained in RPMI-1640 medium, supplemented with 10% heat-inactivated fetal bovine serum and 1% penicillin–streptomycin (Sigma-Aldrich, St Louis, MO, USA). Braunschweig, Germany). ShRNA sequence targeting NALT was cloned into lentivirus vector U6-MCS-Ubiquitin-Cherry-IRES-puromycin while the overexpression of NICD was conducted by cloning the coding sequence (CDS) into lentivirus vector PLv-GFP. The γ secretase inhibitors N-[N-(3, 5-difluorophenacetyl)-l-alanyl]-S-phenylglycine t-butyl ester (DAPT) were purchased from Selleck Chemicals (Houston, TX, USA) which was refer in the figures as GSI treated group. Sequence for NALT shRNA: 5′- CACCGGACTACTTCTCGTTTGAAAGCGAACTTTCAAACGAGAAGTAGTCC –3. Sequence for the ASO targeting NALT: 5′ G*C*T*T*C*C*C*T*C*C*T*A*C*T*T*G*C*C*A*G 3′ and the control sequence: 5′ G*C*C*C*A*T*T*C*A*T*T*T*C*C*T*T*C*C*C*G 3′. The stable cells infection was selected by puromycin. After cells treated with lentivirus for 48 h, medium containing puromycin was added and the medium was replaced every 2 days. The primer antibody of NOTCH1, HES1, MAML1, Survivin and GAPDH were purchased from Cell Signal Technology (Boston, MA, USA).

### Quantitative real time polymerase chain reaction (qRT-PCR)

Quantitative real time polymerase chain reaction (qRT-PCR) was conducted to investigate the expression levels of NALT and NOTCH1 in human samples. Total RNA was obtained from tissues using TRIzol reagent as described by the manufacturer (Invitrogen Life Technologies Co, CA, USA). QRT-PCR was performed using ABI Prism 7900HT (Applied Biosystems, CA, USA) according to the direction of the reagents. The subcellular location of NALT of conducted by using SurePrep™ Nuclear/Cytoplasmic RNA Purification Kit (Thermo Fisher, CA, USA) according to the manufacturer’s protocol. HPRT was used as the control for cytoplasmic expression and U2 for cytonuclear expression.

### Western blot

Protein extracts were electrophoresed on 10% SDS polyacrylamide gels and transferred to nitrocellulose membranes. Membranes were blocked with 5% non-fat dried milk and incubated for overnight with an appropriate primary antibody, followed by the horseradish peroxidase-conjugated secondary antibody. The immunocomplexes were visualized using the chemiluminescence phototope-horseradish- peroxidase kit. GAPDH was used to ensure equivalent protein loading. The integrated density of the band was quantified by ImageJ software.

### Immunocytochemical assay

Cells were fixated with methanol following by the culturing in poly-L-lysine coated microslide as cell smear. The slides were drying for one hour, and then were treated with 3% H2O2 in methanol for 30 min to block the endogenous peroxidase activity. The sections were rinsed in phosphate-buffered saline (PBS) twice, 5 min each time and incubated with 10% normal goat serum for 30 min to block non-specific antibody binding. After washing, the samples were incubated with primary anti-rabbit antibody NOTCH1 at 4 °C overnight, and then washed in PBS for three times and then incubated with secondary antibodies. After that, the sections were stained with DAB according to manufacturer’s protocols and mounted and photographed using a digitalized microscope camera (Nikon, Tokyo, Japan).

### Cell proliferation assay

Cell proliferation was assayed using CCK8 and 5-ethynyl-2′-deoxyuridine, also known as EDU(Roche, Basel, Switzerland) according to the instruction of manufacture.

### Xenograft model

NOD/SCID mice (4–5 weeks old, Shanghai Laboratory Animal Center, Shanghai, China) were housed and bred in a specific pathogen-free animal facility, treated in accordance with the European Union guidelines and approval of the Institutional Ethical Committee of Instituto de Medicina Molecular. Three weeks later, mice were subcutaneously injected with 1 × 10^7^ Jurkat cells. Cells were stably expressed with either NALT shRNA, NICD, GSI, or the control tagged with luciferance. At day 5, mice were injected with luciferin to assess tumor burden by whole-body bioluminescence imaging. For bioluminescence imaging, mice were anesthetized, intraperitoneally injected with 1.5 mg luciferin/g and scanned with an IVIS Lumina bio-imaging device (Caliper Life Sciences, Hopkinton, MA, USA), after 15 min, total flux (photons per second) was calculated using Living Image software (Caliper Life Sciences). For caliper measurements, the Tumor volumes were calculated as length×width^2^ × 0.5.

### Side-population cell detection and sorting

Cells were incubated with the transport blockers verapamil (100 μM; Sigma-Aldrich, MO, USA), 20 minutes prior to the Hoechst 33342 incubation. Propidium iodide (2 μg/ml; Sigma-Aldrich, MO, USA) was added to exclude dead cells. The cell suspensions were analysed using a FACSAriaII (BD Biosciences, CA, USA). The SP was visualized after UV excitation on the basis of blue emission through a 424/44 filter and of red emission through a 630/22 filter (Omega Optical, Brattleboro, VT). Within the living cell population (propidium iodide negative), the side and main population (MP) were sorted separately and collected. Immunocytochemistry was performed on the SP cells to assess NOTCH1 expression in the sorted samples.

### Gal4-λN/BoxB reporter assay

In this system, the BoxB RNA stem loop is fused to NALT, LUNAR1 was used as a positive control as described previously^38^. The plasmid encoding a TK-luciferase gene under the control of five GAL4 UAS sites was co-transfected with plasmids encoding GAL4-λN peptide fused to a C-terminal GFP tag, BoxB as described above. Ranilla luciferase was regarded as control in this system. The binding of Gal4-λN fusion was confirmed firstly.

### ASO technology

Antisense oligonucleotides (ASOs) were designed using the IDT Antisense Design Tool (http://www.idtdna.com) using the chimeric 25-mer setting. The top 3 ASOs generated by the design tool were ordered and tested for knockdown efficiency for further investigation. Sequence for the control group in ASO assay was taken from Thomas Trimarchi, *et al*.^38^. For ASO experiments in T-ALL cells oligonucleotides were delivered simply by adding them to the growth media at 2 μM. For ASO knockdown in BoxB tethering experiments, ASOs were co-transfected with plasmid DNA at 50 nM.

### Statistical analysis

The results of qRT-PCR were presented the mean (standard error) and the student’s t-test analysis of variance was used to evaluate statistical differences in demographic characteristics. Pearson correlation analysis was used to analyze the relationship of expression level of clinical samples between NALT and NOTCH1 in patients. Statistical analysis was performed using STATA 9.2, and presented with Graph PAD prism software. P < 0.05 was considered significant throughout the study.

## Additional Information

**How to cite this article**: Wang, Y. *et al*. LncRNA NALT interaction with NOTCH1 promoted cell proliferation in pediatric T cell acute lymphoblastic leukemia. *Sci. Rep*. **5**, 13749; doi: 10.1038/srep13749 (2015).

## Supplementary Material

Supplementary Information
