# Supplementary material for: LncRNA NALT interaction with NOTCH1 promoted cell proliferation in pediatric T cell acute lymphoblastic leukemia
Source: Sci Rep. 2015 Sep 2;5:13749. doi: 10.1038/srep13749 (PMC4557127; doi:10.1038/srep13749)
Supplement: Supplementary Information [file srep13749-s1.pdf]

Supplementary Information

LncRNA NALT interaction with NOTCH1 promoted cell proliferation in pediatric T cell acute lymphoblastic leukemia

Running Title: NALT in pediatric T-ALL

Yaping Wang[1, §], , Peng Wu[1, §], Lulu He[1, §], Liucheng Rong, Yao Xue, Yongjun Fang[1, *]

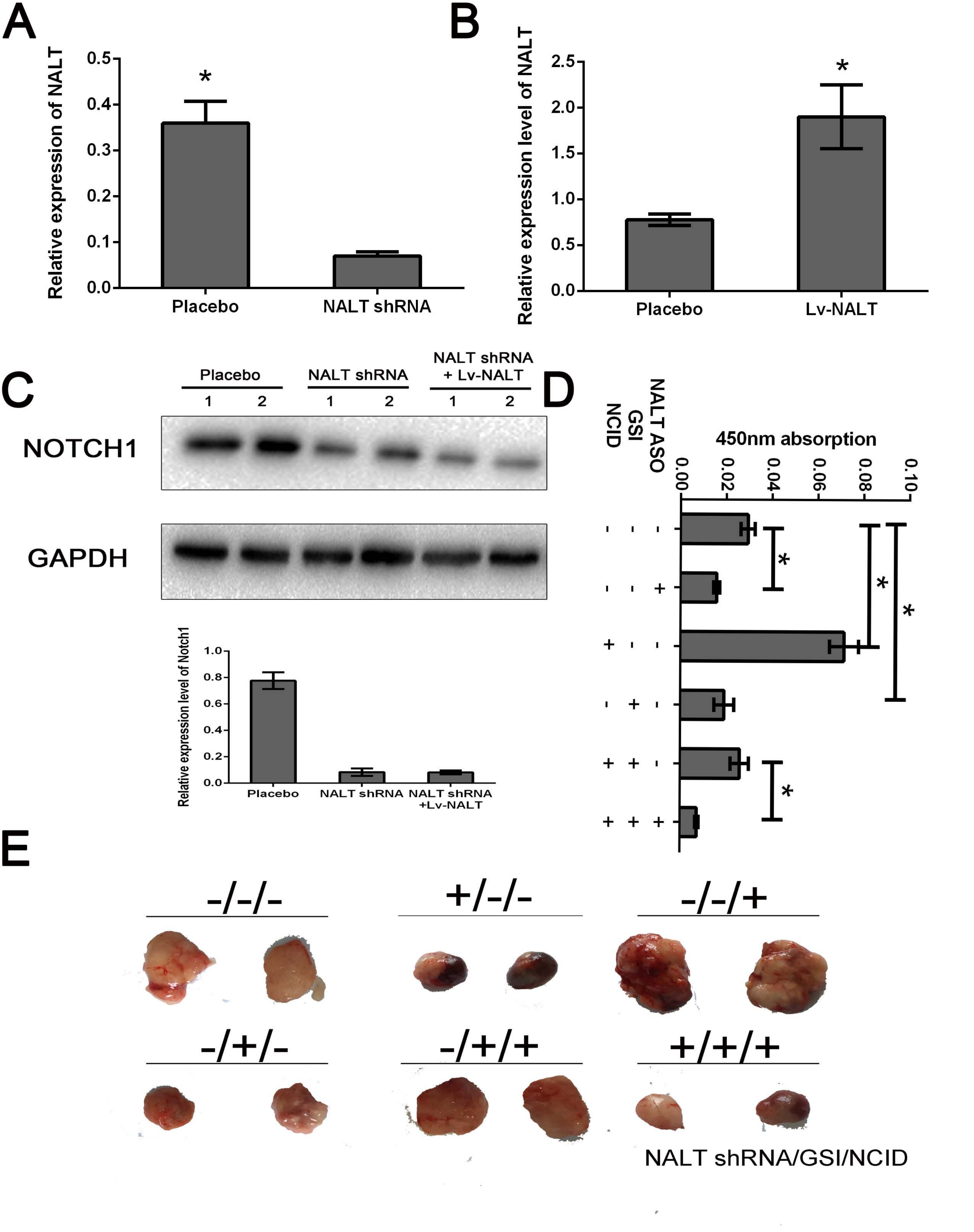

A
Relative expression of NALT
0.5
0.4
0.3
0.2
0.1
0.0
*
Placebo
NALT shRNA
B
Relative expression level of NALT
2.5
2.0
1.5
1.0
0.5
0.0
*
Placebo
Lv-NALT
C
Placebo
NALT shRNA
NALT shRNA
+ Lv-NALT
1
2
1
2
1
2
NOTCH1
GAPDH
Relative expression level of Notch1
1.0
0.8
0.6
0.4
0.2
0.0
Placebo
NALT shRNA
NALT shRNA
+Lv-NALT
D
NALT ASO
GSI
NCID
450nm absorption
0.00
0.02
0.04
0.06
0.08
0.10
- - -
- - +
+ - -
- + -
+ + -
+ + +
*
*
*
*
E
-/-/-
+/-/-
-/-/+
-/+/-
-/+/+
+/+/+
NALT shRNA/GSI/NCID

Supplementary Figure legends

Supplementary Figure S1. Decreased NOTCH1 induced by NALT shRNA could not rescued by ectogenous NALT.

A: The down-regulation of NALT by NALT shRNA was confirmed by RT-PCR. B: The over-expression of NALT by lentivirus was confirmed by RT-PCR. C: The mRNA and protein expression of NOTCH1 in NALT shRNA cells co-treated with ectogenous NALT. D: CCK8 assay was conducted by using ASO targeting NALT. E: The volume of each tumor was calculated as the length $\times$width^2 $\times$0.5. The tumor was obtained after 35 days.
